# To what extent is the variation in cardiac rehabilitation quality associated with patient characteristics?

**DOI:** 10.1186/s12913-018-3831-1

**Published:** 2019-01-03

**Authors:** Ahmad Salman, Patrick Doherty

**Affiliations:** 1 Department of Health Sciences, University of York, York, UK; 2 Ministry of Health, Kuwait City, Kuwait

**Keywords:** Cardiac rehabilitation, Quality of care and outcomes, Delivery of care, Observational study

## Abstract

**Background:**

Huge variability in quality of service delivery of cardiac rehabilitation (CR) in the UK. This study aimed to ascertain whether the variation in quality of CR delivery is associated with participants’ characteristics.

**Methods:**

Individual patient data from 1 April 2013 to 31 March 2014 were collected electronically from the UK’s National Audit of Cardiac Rehabilitation database. Quality of CR delivery is categorised as low, middle, and high based on six service-level criteria. The study included a range of patient variables: patient demographics, cardiovascular risk factors, comorbidities, physical and psychosocial health measures, and index of multiple deprivation.

**Results:**

The chance that a CR patient with more comorbidities attended a high-quality programme was 2.13 and 1.85 times higher than the chance that the same patient attended a low- or middle-quality programme, respectively. Patients who participated in high-quality CR programmes tended to be at high risk (e.g. increased waist size and high blood pressure); high BMI, low physical activity levels and high Hospital Anxiety and Depression Scale scores; and were more likely to be smokers, and be in more socially deprived groups than patients in low-quality programmes.

**Conclusions:**

These findings show that the quality of CR delivery can be improved and meet national standards by serving a more multi-morbid population which is important for patients, health providers and commissioners of healthcare. In order for low-quality programmes to meet clinical standards, CR services need to be more inclusive in respect of patients’ characteristics identified in the study. Evaluation and dissemination of information about the populations served by CR programmes may help low-quality programmes to be more inclusive.

## Background

Cardiovascular disease (CVD) remains the leading cause of death globally and is one of the most common causes of long-term disability [1]. One in three deaths worldwide are the result of CVD, yet most cases are preventable. Cardiovascular disease is also a major contributor to health inequity in the United Kingdom (UK) [2].

Cardiac rehabilitation (CR) is a multicomponent intervention that targets risk factors and psychosocial wellbeing and that is delivered by skilled multidisciplinary teams [3]. Mounting evidence from robust trials and registry data indicate that CR is clinically beneficial and cost effective, with multifaceted secondary prevention services resulting in decreased cardiovascular morbidity and mortality in patients with CVD [4–8]. Cardiac rehabilitation is also recommended by the European Association of Preventive Cardiology and the British Association for Cardiovascular Prevention and Rehabilitation (BACPR) [3, 9].

The British Heart Foundation’s National Audit of Cardiac Rehabilitation (NACR) collects data on service delivery and patient outcomes [10]. In 2015, the BACPR and NACR developed the National Certification programme for Cardiovascular Rehabilitation (NCP_CR) which set out to improve delivery of CR, showcase good services, and seek to ensure the effectiveness of routine provision of CR programmes through achievement of a minimum level of service delivery across the UK [3, 10, 11]. Cardiac rehabilitation in the UK is delivered to groups of patients in healthcare or community centres using a mixture of supervised or unsupervised approaches conducted in any setting (inpatient, outpatient, community, home based) [12]. Most CR programmes comprise weekly attendance at group sessions for an average of 63 days or 9 weeks [10].

Despite the strong evidence-based standards for service delivery of CR, it has become apparent from recent NACR reports and journal publications that CR is not delivered equitably across the UK [10, 13]. There are differences at programme level either defined by NCP_CR or local variation [10, 13]. However, the role played by patient characteristics in associating whether the quality of delivery of CR services is high, medium, or low remains unclear [10, 13]. Moreover, continued debate in the literature suggests that some CR programmes are suboptimal in terms of delivery, are less effective, and might not achieve expected outcomes [12, 14–16]. It is important to investigate whether the populations attending CR programmes are the same across the three quality categories of CR. The aim of this study was to ascertain whether the variation in CR quality is associated with the participating patients’ characteristics.

## Methods

This study follows the Strengthening the Reporting of Observational Studies in Epidemiology (STROBE) guidelines [17].

### Data collection

Analyses were conducted using individual patient data collected electronically in the NACR database from 1 April 2013 to 31 March 2014; this relates to the first year in which the NCP-CR minimum performance criteria, which use national averages reported by the NACR, were in place to audit the delivery of CR [11, 18]. National Health Service (NHS) Digital has approval to collect patient-identifiable data which are then removed before any data extract made available to the NACR team [19]. This data governance process removes the need for explicit consent from individual patients for the purposes of audit and service related research under Section 251 of the NHS Act 2006. The audit is voluntary and supports direct entry of data within a secure online system, collecting local programme-level data on the delivery of CR alongside patient-level data for those who are referred to and undergo CR. Only clinically approved users, verified through a Caldicott Guardian, are able to input data. This includes details of a patient’s initiating event, type of treatment, risk factors, medications, demographics, and clinical outcomes before and after CR. NACR data governance approval is reviewed annually by NHS Digital. So long as the data is used for valid NACR purposes and works within agreed data protection processes then separate ethical approval is not required. Patients were included in our study if they started CR, had been assessed at baseline, and had follow-up data at an assessment after CR.

### Procedure

This study used the methods reported in a previous study [13], to categorise the quality of delivery of CR programmes as low, middle, and high based on six service-level criteria. The previous study was designed to assess the extent by which CR programmes met six service-level NCP_CR measures deemed important for the delivery of high-quality rehabilitation: (1) whether CR is delivered to at least four priority groups, (2) assessing before and (3) after CR, and (4) whether rehabilitation of sufficient duration is delivered (5) in a timely manner for myocardial infarction or percutaneous coronary intervention or (6) coronary artery bypass surgery patients [13]. Our study used a variety of different patient variables collected by the NACR [18], including demographic characteristics, cardiovascular risk factors, comorbidities, and physical and psychosocial health measures. To evaluate the role of social deprivation, the study included the English Index of Multiple Deprivation (IMD), which linked to the NACR. The IMD is an overall relative measure of deprivation constructed by combining seven domains of deprivation—income; employment; education, skills and training; health and disability; crime; barriers to housing and services; and living environment—according to their respective weights [20]. The IMD provided Lower Layer Super Output Areas (LSOA) and published deprivation ‘deciles’ alongside ranks [20]. These deciles are calculated by ranking the 32,844 sub-areas in England from the most deprived to the least deprived and dividing them into 10 equal groups [20]. These groups are ranked from 1 to 10, where 1 means that that the LSOA is among the most deprived 10% nationally and 10 represents the least deprived 10%.

Risk stratification published by the BACPR is a multi-factorial measure used to establish prognosis of future major cardiac events or exercise complications by utilising all relevant patient information (e.g. left ventricular ejection fraction, history of arrhythmia, symptoms, functional capacity) to determine the level of exercise intensity prescribed and supervision required [3, 21]. High-risk patients were defined as those at high-risk for cardiovascular events during exercise training. Adaptations should be made to the CR programme according to their risk stratification and comorbidities. Given the level of risk stratification, high risk patients need rigorous individual assessment and risk profiling to be coupled with appropriate monitoring and a safe management and delivery system when undertaking exercise training [21].

### Statistical analysis

Cardiac rehabilitation programmes were aggregated to identify those that met the minimum NCP_CR criteria. The quality of delivery of CR programmes was categorised into three groups: scores of 5–6 represented high quality, scores of 3–4 represented middle quality, and scores of 1–2 represented low quality. Baseline comparisons between the three categories of CR delivery quality were analysed using a one-way analysis of variance (ANOVA) for continuous variables. Effect sizes are reported as partial eta squared (η^2^). Two multinomial logistic regression models were used to test for independent predictors of high quality in the delivery of CR, using the high quality category as the reference. Variables were considered in the models according to their association with the three CR quality categories [22], so cardiovascular risk factors such as body mass index (BMI) and comorbidity variables such as the mean number of comorbidities and the proportion of patients with diabetes, stroke and asthma were considered in the final model to address the aim of identifying higher level quality criteria in the delivery of CR. A *p* value ≤0.05 was considered statistically significant. All data were analysed using IBM SPSS Statistics 24 (New York, USA).

## Results

A one-way ANOVA was conducted to determine whether the mean value of each of the baseline patient characteristics differed between categories of delivery quality (Table 1). No significant differences in age, gender, employment status or IMD were noted between the three categorises. In the high-quality programmes, patients at baseline tended to be in the most deprived 10% nationally compared to those in the low- and middle-quality programmes.

**Table 1:** Demographics and baseline health states of patients in cardiac rehabilitation (CR) programmes classified as having low-, middle- and high-quality service delivery

	Quality category			*p* Value	Effect size
	Low (N)	Middle (N)	High (N)		
Demographics					
Age (years)	63.94 (31)	64.25 (78)	64.64 (52)	0.33	0.01
Female (%)	25.64 (30)	26.01 (77)	26.89 (52)	0.59	0.01
Unemployment (%)	15.96 (20)	19.27 (65)	17.78 (48)	0.56	0.01
IMD (mean)	6.23 (24)	5.90 (66)	5.86 (46)	0.57	0.01
Baseline health state					
High risk (%)	16.28 (12)	21.84 (53)	23.39 (45)	0.32	0.02
BMI (kg/m2) (mean)	27.49 (30)	28.02 (77)	28.39 (51)	0.04*	0.04
Waist (cm) (mean)	97.47 (25)	98.07 (63)	101.00 (44)	0.44	0.01
BP 140 / 90 (%)	28.69 (25)	32.64 (70)	33.47 (52)	0.24	0.02
Smoker (%)	8.32 (18)	12.68 (60)	11.39 (48)	0.09	0.04
6MWT (metres) (mean)	342.74 (13)	276.66 (27)	280.61 (25)	0.15	0.06
ISWT (metres) (mean)	374.58 (7)	326.18 (30)	352.33 (25)	0.62	0.02
150 min moderate/week (%)	36.49 (31)	28.04 (76)	29.53 (52)	0.12	0.03
75 min vigorous/week (%)	8.38 (30)	6.20 (76)	6.56 (52)	0.31	0.02
HADS anxiety (%)	28.05 (18)	32.58 (68)	31.54 (50)	0.16	0.03
HADS depression (%)	18.24 (17)	21.89 (66)	21.69 (50)	0.22	0.02

N, number of CR programmes; %, proportion of patients; *BP* blood pressure, *BMI* body mass index, *HADS* Hospital Anxiety and Depression Scale, *IMD* Index of Multiple Deprivation, *ISWT* Incremental Shuttle Walk Test, *6MWT* Six-Minute Walk Test. %, proportion of patients, *BP* blood pressure, *BMI* body mass index, *HADS* Hospital Anxiety and Depression Scale, *ISWT* Incremental Shuttle Walk Test, *6MWT* Six-Minute Walk Test

*p ≤ 0.05

One-way ANOVA was also conducted to determine whether there were differences in baseline health status between the three categories of service delivery (Table 1). Body mass index differed significantly among the categories. The CR programmes with high-quality delivery included more patients at higher risk—higher BMI and waist circumference, high blood pressure (> 140/90 mmHg), more patients who smoke, and more severe anxiety or depression—at enrolment than low-quality programmes. Patients in high-quality programmes also had poorer physical capacity and lower self-reported physical activity status at baseline.

One-way ANOVA of the proportion of patients with a comorbidity at baseline showed that the mean total of comorbidities and proportion of patients with both diabetes and claudication differed significantly between the three service delivery performance categories (Table 2).

**Table 2:** Baseline comorbidity profiles of patients in cardiac rehabilitation (CR) programmes classified as having low-, middle- and high-quality service delivery

Comorbidity	Quality category			*p* Value	Effect size
	Low (31)	Middle (78)	High (52)		
Total comorbidities (mean)	1.36	1.44	1.72	0.05*	0.04
Angina %	12.23	12.07	14.38	0.43	0.01
Arthritis %	7.94	9.72	11.42	0.13	0.03
Cancer %	2.99	4.32	4.73	0.10	0.03
Diabetes %	9.99	13.93	15.90	0.01*	0.06
Rheumatism %	1.77	1.61	2.09	0.43	0.01
Stroke %	2.07	3.28	3.79	0.01*	0.06
Osteoporosis %	1.08	1.27	1.86	0.05	0.04
Hypertension %	31.89	31.87	35.58	0.47	0.01
Chronic bronchitis (COPD) %	1.22	3.04	2.55	0.54	0.01
Emphysema %	0.53	1.50	1.80	0.13	0.03
Asthma %	4.59	4.65	6.55	0.01*	0.06
Claudication %	3.07	1.47	2.41	0.19	0.02
Chronic back problems %	5.02	6.58	8.33	0.12	0.03
Anxiety %	4.78	1.96	2.82	0.29	0.02
Depression %	4.92	2.73	3.20	0.48	0.01
Family history of CVD %	9.58	11.28	11.69	0.74	0.00
Hypercholesterolaemia or dyslipidaemia %	17.21	15.58	18.74	0.56	0.01

% proportion of patients, *COPD* chronic obstructive pulmonary disease, *CVD* cardiovascular disease

*p ≤ 0.05

Tables 3 and 4 outlines the results of the two multinomial logistic regression models. We included all baseline parameters that were statistically significant according to ANOVA. The first regression was performed to ascertain the effects of BMI and mean number of comorbidities at baseline on the likelihood that programmes categorised as high quality.

**Table 3:** Multinomial regression models for independent predictors of category of quality for CR delivery

Measured variables	b (SE)	Lower	Odds ratio	Upper
Low- vs high-quality categories				
Intercept	11.86 (4.69)*			
Mean total comorbidities	−0.76 (0.34)*	0.24	0.47	0.90
BMI	−0.40 (0.17)*	0.49	0.67	0.93
Middle- vs high-quality categories				
Intercept	7.71 (4.15)			
Mean total comorbidities	−0.62 (0.26)*	0.32	0.54	0.90
BMI	−0.22 (0.15)	0.60	0.80	1.06

*BMI* body mass index, *b* regression coefficients, *SE* standard error

*p ≤ 0.05

**Table 4:** Multinomial regression models for independent predictors of category of quality for CR delivery

Measured variables	b (SE)	Lower	Odds ratio	Upper
Low- vs high-quality categories				
Intercept	1.19 (0.52)*			
Diabetes (%)	−0.09 (0.05)*	0.83	0.91	0.99
Stroke (%)	−0.24 (0.13)	0.62	0.79	1.01
Asthma (%)	0.03 (0.09)	0.87	1.03	1.23
Middle- vs high-quality categories				
Intercept	1.27 (0.44)*			
Diabetes (%)	−0.01 (0.03)	0.95	1.00	1.05
Stroke (%)	0.05 (0.09)	0.88	1.05	1.25
Asthma (%)	−0.17 (0.07)*	0.74	0.84	0.96

*%* proportion of patients, *b* regression coefficients, *SE* standard error

*p ≤ 0.05

The model was based on 158 CR programmes (Low = 30, middle = 77, High = 51) with complete data. The model was statistically significant: χ^2^(4) = 14.05 (*p* = 0.01). Of the two predictor variables, both were statistically significant (Table 3).

Increasing mean BMI and number of total comorbidities were associated with a decrease in the odds of being in the low-quality service delivery category compared to high category, with an odds ratio of 0.67 (95% Confidence Interval (CI) (0.49, 0.93)) and 0.47 (95% CI (0.24, 0.90)), respectively.

The second regression was performed to ascertain the effects of components of comorbidities: proportion of patients with diabetes, stroke and asthma at baseline on the likelihood that that programmes categorised as high quality. The model was based on the 161 CR quality programmes (Low = 31, middle = 78, High = 52) with complete data. The model was statistically significant, χ2(6) = 24.79 (*p* < 0.001). Of the three predictor variables, two were statistically significant: proportion of diabetes comorbidity and proportion of asthma comorbidity (Table 4).

Increasing mean proportion of patients with diabetes comorbidity was associated with a decrease in the odds of being in the low-quality compared to high category, with an odds ratio of 0.91 (95% CI (0.83, 0.99)). An increase in mean proportion of patients with asthma comorbidity was associated with a decrease in the odds of being in the middle-quality compared to high category, with an odds ratio of 0.84 (95% CI (0.74, 0.96)).

## Discussion

There were significant differences in the patient population between the quality categories for delivery of CR services. Previous research that examined the quality of delivery of CR programmes in the UK identified three distinct categories and proportions—low (30.6%), middle (45.9%), and high (18.2%) of CR quality [13]. We investigated whether the three quality categories differed with regard to the populations being treated within them. A CR programme was more likely to be categorised as high quality if it included patients with a higher mean total of comorbidities, including diabetes, stroke, and asthma in addition to high BMI.

According to our findings, high-quality programmes recruit more patients with multiple comorbidities, who are more representative of the broader CVD population than those with few comorbidities. The presence of multiple comorbidities including stroke, diabetes, chronic obstructive pulmonary disease is an important factor associated with a lower likelihood of a patient being referred to and participating in CR [23–26], and the authors of a systematic review warned that CR programmes need to pay greater attention to recruitment of patients with multiple morbidities [4]. However, patients with multiple morbidities represent populations at significantly increased cardiovascular risk who may benefit from the services provided in CR [23–26].

For one additional comorbidity, the odds of being in the high-quality service increases by a factor of 2.13 as opposed to low quality and by a factor of 1.85 as opposed to middle quality, which indicates that high-quality CR programmes take on more complicated cases and potentially higher risk patients than low or middle-quality programmes. The presence of multiple comorbidities is an important factor associated with lower odds of referral to, participation in, and uptake of CR [23–26]. The high-quality CR programmes included more patients with the most dominant morbidities associated with CVD according to the NACR [10]— hypertension, hypercholesterolaemia or dislipidaemia, diabetes, angina, combination of respiratory conditions (chronic bronchitis, emphysema, and asthma), arthritis, chronic back problems, cancer, stroke,—at entry to CR than the low-quality programmes.

For each unit increase in the BMI, the odds of being in the high-quality category increases by a factor of 1.49 as opposed to low quality. Obesity is an independent risk factor for the development of CVD [27] and higher BMI was associated with shorter longevity and significantly increased risk of cardiovascular morbidity and mortality compared with normal BMI [28]. At entry into CR, more than 80% of patients are overweight and 30% have BMI > 30 kg/m^2^ [10, 29]. Cardiac rehabilitation programmes do not generally include weight-loss components [29], but CR programmes with high-quality delivery recruit more patients with CVD and higher BMI than those with low-quality delivery.

For each percent increase in the proportion of patients with diabetes comorbidity, the odds of being in the high-quality category increases by a factor of 1.10 as opposed to low quality. Despite the fact that CVD is the most prevalent cause of mortality and morbidity in diabetic populations [30] and in addition to the fact that patients with diabetes had more CVD risk factors and lower physical capacity than patients without diabetes at the beginning of CR [30, 31], the findings show that high-quality programmes recruit more patients with CVD and diabetes than low-quality programmes. Previous studies have examined the benefit of CR in diabetes [32, 33]. Cardiac rehabilitation patients with diabetes comorbidity emphasizes the need to target diabetic patients in CR programs for an aggressive program of risk factor management [31]. The prevalence of diabetic patients in CR programs appears to be increasing, and is likely to continue to rise as the current trends indicating increase of prevalence of diabetes [31]. Diabetic patients are more depressed following CVD and have lower scores for functional status, well-being, and total quality of life than non-diabetic patients [34]. Cardiac rehabilitation in diabetic patients results in marked reduction in depression to a prevalence rate identical to non- diabetic patients in addition to improvements in exercise capacity and total quality of life following CR [34].

For each percent increase in the proportion of patients with asthma comorbidity, the odds of being in the high-quality category increases by a factor of 1.19 as opposed to middle quality. The findings show that high-quality programmes recruit more patients with CVD and asthma than low- and middle- quality programmes. Asthma is one of the global morbidity and is the most common chronic respiratory diseases worldwide and it was prospectively associated with increased risk of major CVD [35, 36]. Recent meta-analysis results indicate that asthma was associated with an increased risk of CVD and all-cause mortality in cohort studies [37]. Large cohort studies provide more evidence that asthmatics have a higher CVD event rates and an increased risk of death than non-asthmatics [38, 39]. Comorbidity asthma was associated with a decreased likelihood of CR attendance among cardiac patients [40].

The results of the analysis of social deprivation showed no statistically significant difference in social deprivation among quality categories, high-quality programmes tended to recruit more socially deprived patients than low- and middle-quality programmes. Previous studies suggested that socioeconomic deprivation is associated with lower participation in CR, as non-participants tend to be more socially deprived [41–43]. A systematic review showed that patients with greater deprivation are less likely to attend CR programmes but may have the most to gain from CR because of a linear relation between socioeconomic status and cardiac outcomes [44].

Patients who participated in high-quality CR programmes tended to be those with high-risk status, high BMI score, high waist circumference, and high blood pressure, high HADS anxiety and depression score, smokers; to have more comorbidities; and to be in more socially deprived groups than patients in the low-quality programmes. In addition, high-quality CR programmes also take on patients with lower fitness levels than low-quality programmes. Such patients often have more severe functional impairment and are most in need of CR, as well as being most likely to benefit [45].

Ensuring equity of access to CR and improving the consistency of delivery should increase long-term behaviour changes and contribute to a reduction in CVD-related health inequality [46]. The data analysis shows that there are significant differences between low-, middle and high quality of CR programme in staffing or number of qualified multidisciplinary team (MDT) as a surrogate for well-resourced programmes. 63% of CR programmes in the low-quality programmes comprise of at least three different professions in the CR team while 73.7 and 85.4% of middle and high quality programmes delivered by MDT (3+) respectively.

Although the BACPR recommends staffing to be multi-disciplinary [3], some CR programmes have varying staffing and less physical resource (equipment and location space) which can impact on patient recruitment. In addition, around 20% of CR programmes don’t carry out formal assessment at baseline which again may influence the type of patients they receive [10]. Patient choice is a reality in the UK where patients can ask to be referred to a CR programme not associated with their local hospital.

This is the only UK-specific study to ascertain whether the variation in quality of CR delivery is, in-part, determined by the patient characteristics, while also addressing whether these differences are associated with better quality delivery. This study accounted for the range of patients within programmes in terms of demographic characteristics, cardiovascular risk factors, comorbidities, and physical and psychosocial health measures collected by the NACR. Evaluation and dissemination of information about the populations served by CR programmes may help low-quality programmes to be more inclusive.

### Strengths and limitations of this study

The use of an observational approach based on voluntary and routinely collected patient data is a strength in respect of real-world representation. According to the 2017 NACR report [10], only 224 of the 303 CR programmes in the UK entered data electronically to the NACR. It can be argued that this provided enough data to be representative and to carry out a reliable analysis, but future studies should aim to achieve greater capture of available patient records across the UK.

## Conclusions

This study aimed to ascertain whether the variation in quality of CR delivery is associated with patients’ characteristics. Mean total comorbidities, higher BMI scores, proportion of patients with diabetes or asthma were associated with CR programmes categorised as high quality. This finding shows that the quality of delivery of a CR programme is associated with the morbidity profile of its patient population. The quality of CR delivery can be improved and meet national standards by serving a more multi-morbid population which is important for patients, health providers and commissioners of healthcare. In order for low-quality programmes to meet clinical standards, CR services need to be more inclusive in respect of patients’ characteristics identified in the study. Further research is required to investigate the extent of patient outcomes between high-quality, middle quality and low-quality CR programmes in addition to investigate CR programmes’ characteristics and the impact of program location on quality.

## Abbreviations

%: Percentage/Proportion
BACPR: British Association for Cardiovascular Prevention and Rehabilitation
BHF: British Heart Foundation
BMI: Body mass index
BP: Blood pressure
CI: Confidence interval
CVD: Cardiovascular disease
MDT: Multidisciplinary team
NCP_CR: National Certification Programme for Cardiovascular Rehabilitation
NHS: National Health Service
NICE: National Institute for Health and Care Excellence
SE: Standard error
UK: United Kingdom
